# Exogenous Dopamine Alleviates Combined High Temperature and Drought Stress in Loquat [*Eriobotrya japonica* (Thunb.) Lindl.] Seedlings: Improvements in Photosynthetic Efficiency, Oxidative Damage and Osmotic Regulation

**DOI:** 10.3390/plants14172650

**Published:** 2025-08-26

**Authors:** Xian Luo, Ya Luo, Xiao-Li Wang, Xiao-Mei Kong, Hui-Fen Zhang, Li-Jin Lin, Yu-Xing Li, Ke-Wen Huang, Qun-Xian Deng, Yong-Xia Jia

**Affiliations:** 1 College of Horticulture, Sichuan Agricultural University, Chengdu 611130, China; 13996@sicau.edu.cn (X.L.); 13595173483@163.com (Y.L.); wangxiaoli9264@163.com (X.-L.W.); 2024305077@stu.sicau.edu.cn (X.-M.K.); hfcheung@sicau.edu.cn (H.-F.Z.); 14208@sicau.edu.cn (L.-J.L.); lyx0707edu@163.com (Y.-X.L.); dengqx@sicau.edu.cn (Q.-X.D.); 2 Horticultural Research Institute, Chengdu Academy of Agriculture and Forestry Sciences, Chengdu 611130, China; hkewen@foxmail.com; 3 College of Resources, Sichuan Agricultural University, Chengdu 611130, China

**Keywords:** combined high temperature and drought stress, dopamine, loquat, photosynthetic characteristics, oxidative damage, osmotic adjustment substance

## Abstract

In recent years, high temperature and drought have severely impacted the growth and development of loquat [*Eriobotrya japonica* (Thunb.) Lindl.] plants. Although dopamine can improve the stress resistance of plants, its role in combined stress requires further exploration. This study investigated the alleviative effect and mechanism of exogenous dopamine on loquat seedlings subjected to the combined stress of high temperature and drought. The combined stress significantly reduced root viability, photosynthetic pigment content, and net photosynthetic rate (*P*n) while markedly increasing reactive oxygen species (ROS) levels, thiobarbituric acid-reactive substances (TBARS) content, and electrolyte leakage (EL). The seedlings exhibited pronounced wilting symptoms, along with markedly reduced root surface area and volume. Dopamine treatment significantly alleviated combined stress-induced damage. This mitigation was manifested through substantially enhanced root viability, photosynthetic pigment content, *P*n, antioxidant enzyme activities, and osmotic adjustment substances concomitantly with marked reductions in ROS, TBARS content, and EL. Dopamine significantly reduced seedling wilting severity and improved root morphological parameters. This study demonstrates that dopamine enhances loquat seedlings’ tolerance to combined stress through coordinated mechanisms: maintaining photosynthetic pigments and improving stomatal conductance to sustain photosynthetic efficiency, enhancing antioxidant enzyme activity and ROS scavenging capacity to mitigate oxidative damage, and promoting osmotic solute accumulation for osmotic potential regulation.

## 1. Introduction

In recent years, global climate warming has significantly increased the frequency, intensity, and duration of extreme dry-heat events [1,2]. As a synergistic abiotic stress factor, combined high temperature and drought stress poses an increasingly severe threat to agricultural ecosystems [3,4]. Notably, China’s high temperature and drought risk index reached its highest level since 1961 in 2022 [5], indicating that this compound stress poses a substantial risk to the country’s agricultural productivity and sustainable development. 

Loquat [*Eriobotrya japonica* (Thunb.) Lindl.], a Rosaceae family member, ranks among the principal fruit trees in subtropical regions [6]. As both the origin and core distribution area of this species, China accounts for more than 66% of global cultivation area and yield, establishing it as the dominant worldwide producer [7]. Loquat thrives in warm and humid conditions, with optimal growth at 15–30 °C. Exposure to temperatures > 35 °C induces leaf wilting, fruit sunscald, and impaired root development [8]. Owing to its shallow root system, the species exhibits high sensitivity to water availability [9]. The seedling stage is critical for stress tolerance development, as combined high temperature and drought stress during this phase severely constrains mature tree growth and fruit quality formation.

High temperature or drought stress individually impairs photosynthesis, disrupts reactive oxygen species (ROS) homeostasis, and compromises osmotic adjustment in plants. Photosynthesis underpins plant growth, development, and energy acquisition, its efficiency provides a critical gauge of stress-induced impairment to primary productivity [10,11]. Concurrently, imbalanced ROS metabolism is a primary indicator of oxidative damage, effectively reflecting both membrane integrity impairment and oxidative stress severity [11,12]. The accumulation of osmoregulatory solutes constitutes an adaptive mechanism for turgor maintenance and osmotic stress counteraction; variations in solute levels directly signal a plant’s capacity to sustain water and osmotic homeostasis [13]. Mechanistically, high temperature predominantly induces thylakoid membrane disintegration and reduces rubisco activity, thereby decreasing both the net photosynthetic rate (*P*_n_) and the maximum photochemical efficiency of PSII (*F*_v_/*F*_m_) [10,14]. In contrast, drought is the secondary stress induced by high temperature; it triggers stomatal closure, limiting CO_2_ diffusion, and similarly suppresses *P*_n_ [15,16]. Each stress independently induces excessive ROS accumulation, leading to membrane lipid peroxidation and inhibiting osmolyte synthesis (e.g., proline and betaine). This cascade weakens cellular water retention and ultimately curbs dry matter accumulation [9,17,18]. Critically, when high temperature and drought co-occur, they act synergistically, generating effects that substantially exceed the sum of their individual impacts [12].

Applying plant growth regulators represents a promising strategy for enhancing crop stress resilience. Notably, dopamine (DA)—a catecholamine present in animals and plants—mediates dual physiological functions: stress signaling and antioxidant defense [19,20]. Our prior work established that dopamine can improve the activity of antioxidant enzymes and enhance the ability to remove ROS, thereby improving the drought stress tolerance of loquat seedlings [21]. Similar studies have been confirmed in apples [22]. Under high temperature stress, dopamine preserves photosynthetic function by elevating chlorophyll content and *F*v/*F*m while concurrently upregulating antioxidant enzyme genes (e.g., *SOD*, *CAT*, *POD*, and *APX*) to enhance their activities. This dual action synergistically suppresses ROS accumulation, improving thermotolerance [23]. However, current research exclusively focuses on dopamine under single-stress conditions; its physiological roles and regulatory mechanisms under combined stress may differ. These mechanisms remain uncharacterized.

This study investigated photosynthetic characteristics, antioxidant systems, and osmoregulatory substances in loquat seedlings under combined high temperature and drought stress, aiming to explore the (1) mitigation effects of dopamine on combined stress in loquat seedlings and (2) the physiological mechanisms underlying dopamine-mediated tolerance to this combined stress.

## 2. Results

### 2.1. Plant Phenotypes and Root Morphological Parameters of Loquat Seedlings

**Figure 1:**
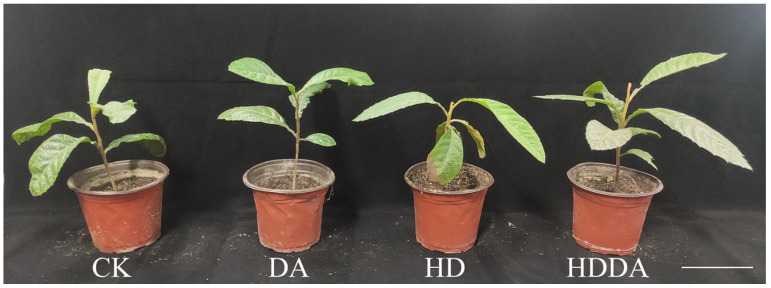
Plant phenotypes of loquat [*Eriobotrya japonica* (Thunb.) Lindl.] seedlings. CK: control, foliar spray with distilled water; DA: foliar spray with dopamine solution; HD: combined high temperature and drought stress, foliar spray with distilled water; HDDA: combined high temperature and drought stress, foliar spray with dopamine solution. Scale bar = 20 cm.

Plant phenotype serves as a visual indicator of damage from combined high temperature and drought stress and dopamine-mediated alleviation. The dopamine (DA) treatment did not induce significant morphological changes in loquat seedlings under normal growth conditions (Figure 1). However, under combined high temperature and drought stress (HD), the seedlings exhibited pronounced wilting symptoms, including leaf drooping, withering, and curling. Notably, the DA application under stress conditions (HDDA) effectively alleviated the growth inhibition caused by HD stress.

As shown in Table 1, no significant differences in root morphological parameters were observed between the CK and DA treatments. Compared with CK, the HD treatment markedly reduced the root surface area, volume, average diameter, and number of tips by 36.30%, 29.69%, 40.04%, 30.21%, and 34.54%, respectively. Relative to HD alone, the HDDA treatment increased these parameters by 45.39%, 8.76%, 30.86%, 22.39%, and 16.11%, respectively.

### 2.2. TWC and RWC of Loquat Seedlings

As shown in Table 2, the DA treatment had no significant effect on the tissue water content (TWC) and relative water content (RWC) of leaves or roots under normal growth conditions. However, the HD treatment significantly reduced the TWC and RWC in both tissues. Compared with CK, the HD treatment decreased the leaf TWC and RWC to 38.02% and 30.79% below control levels and the root TWC and RWC by 19.15% and 25.62%, respectively. Furthermore, the HDDA treatment significantly increased the leaf TWC and RWC by 32.14% and 18.48% and the root TWC and RWC by 13.86% and 18.18% relative to those of the HD treatment alone.

### 2.3. Root Viability, TBARS Content and EL of Loquat Seedlings

**Figure 2:**
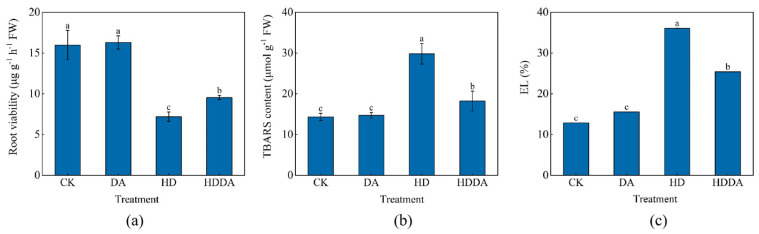
Root viability (**a**), TBARS content (**b**) and EL (**c**) of loquat [*Eriobotrya japonica* (Thunb.) Lindl.] seedlings. Data are presented as the means ± SE (n = 3). Different letters on the bars indicate significant differences (*p* < 0.05) among different treatments based on Tukey’s HSD test. CK: control, foliar spray with distilled water; DA: foliar spray with dopamine solution; HD: combined high temperature and drought stress, foliar spray with distilled water; HDDA: combined high temperature and drought stress, foliar spray with dopamine solution.

Under normal conditions, the loquat seedlings showed no significant differences in root viability, thiobarbituric acid-reactive substances (TBARS) content, or electrolyte leakage (EL) between the DA treatment and the control (Figure 2). Compared with the control plants, the HD-treated plants exhibited a 54.94% decrease in root viability (Figure 2a), while the HDDA treatment resulted in a 32.64% increase compared with the HD treatment. The TBARS content and EL in the HD-treated plants were significantly higher than those in the control (Figure 2b and Figure 2c), with increases of 108.16% and 180.20%, respectively. In contrast, the HDDA treatment reduced both TBARS levels by 38.89% and EL by 29.65% compared with the HD-treated plants.

### 2.4. Photosynthetic Pigment Content and Photosynthetic Parameters of Loquat Seedlings

The carotenoid and total chlorophyll contents in the HD-treated loquat seedlings showed significant decreases of 12.5% and 24.24%, respectively, compared with the control. However, under the HDDA treatment, these pigment levels significantly increased by 42.86% and 22.00%, respectively, compared with those in HD-treated plants.

Compared with CK, the HD-treated loquat seedlings showed significant decreases in the net photosynthetic rate (*P*n), transpiration rate (*T*r), and stomatal conductance (*G*s), whereas the intercellular CO_2_ concentration (*C*i) was significantly increased. Under the HDDA treatment, the *P*n, *T*r, and *G*s values were 2.21, 2.34, and 2.67 times higher than those in the HD-treated plants, respectively. Conversely, the *C*i was significantly reduced by 8.25% compared with the HD-treated plants. (See Table 3).

### 2.5. Antioxidant System of Loquat Seedlings

#### 2.5.1. Superoxide Anion Production Rate and H_2_O_2_ Accumulation

**Figure 3:**
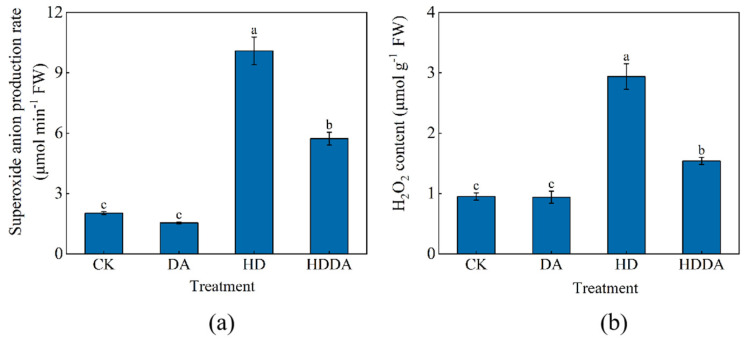
Superoxide anion production rate (**a**) and H_2_O_2_ content (**b**) of loquat [*Eriobotrya japonica* (Thunb.) Lindl.] seedlings. Data are presented as the means ± SE (n = 3). Different letters on the bars indicate significant differences (*p* < 0.05) among different treatments based on Tukey’s HSD test. CK: control, foliar spray with distilled water; DA: foliar spray with dopamine solution; HD: combined high temperature and drought stress, foliar spray with distilled water; HDDA: combined high temperature and drought stress, foliar spray with dopamine solution.

The DA treatment exerted no significant effects on the superoxide anion production rate (Figure 3a) or H_2_O_2_ content (Figure 3b) in the loquat seedlings under normal conditions. However, the HD treatment markedly induced ROS accumulation, increasing the superoxide anion production rate by 398.17% and H_2_O_2_ content by 209.47% compared with CK. In contrast, the HDDA treatment significantly attenuated these stress-induced ROS surges, reducing the superoxide anion production rate by 43.18% and H_2_O_2_ content by 47.62% compared with the HD-treated plants.

#### 2.5.2. Antioxidant Enzyme Activities

**Figure 4:**
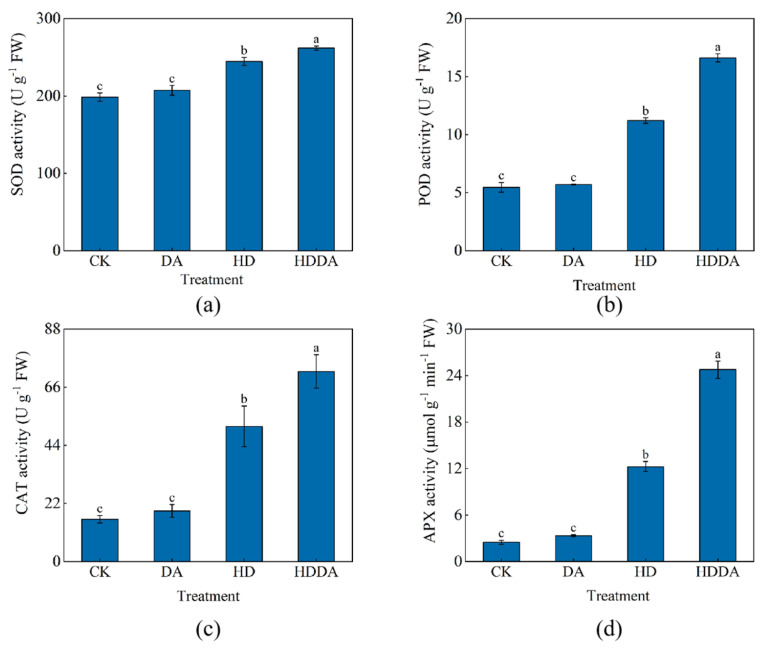
Activities of SOD (**a**), POD (**b**), CAT (**c**), and APX (**d**) of loquat [*Eriobotrya japonica* (Thunb.) Lindl.] seedlings. Data are presented as the means ± SE (n = 3). Different letters on the bars indicate significant differences (*p* < 0.05) among different treatments based on Tukey’s HSD test. CK: control, foliar spray with distilled water; DA: foliar spray with dopamine solution; HD: combined high temperature and drought stress, foliar spray with distilled water; HDDA: combined high temperature and drought stress, foliar spray with dopamine solution.

Figure 4 demonstrates that under the HD treatment, the activities of superoxide dismutase (SOD), peroxidase (POD), catalase (CAT), and ascorbate peroxidase (APX) in the loquat seedlings were significantly higher than those in CK, with increases of 23.28%, 105.12%, 220.00%, and 392.37%, respectively. Compared with the HD treatment, the HDDA treatment further enhanced the activities of these antioxidant enzymes under high temperature and drought stress, showing increases of 7.02%, 48.13%, 40.63%, and 102.04%, respectively. No significant differences in SOD, POD, CAT or APX activities were observed between the DA-treated and CK-treated seedlings under normal conditions.

### 2.6. Osmotic Regulating Substance Accumulation of Loquat Seedlings

**Figure 5:**
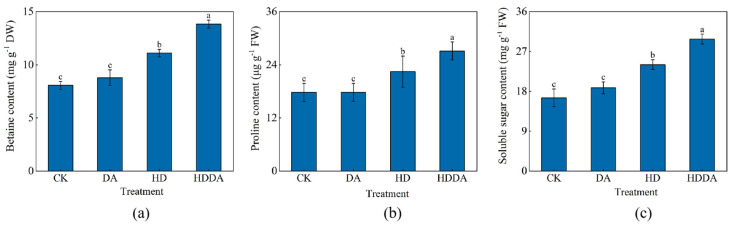
Contents of betaine (**a**), proline (**b**), and soluble sugar (**c**) of loquat [*Eriobotrya japonica* (Thunb.) Lindl.] seedlings. Data are presented as the means ± SE (n = 3). Different letters on the bars indicate significant differences (*p* < 0.05) among different treatments based on Tukey’s HSD test. CK: control, foliar spray with distilled water; DA: foliar spray with dopamine solution; HD: combined high temperature and drought stress, foliar spray with distilled water; HDDA: combined high temperature and drought stress, foliar spray with dopamine solution.

The DA treatment exerted no significant effects on osmotic regulating substance content under normal conditions (Figure 5). Compared with CK, the HD treatment significantly elevated betaine, proline (Pro), and soluble sugar (SS) levels by 37.66%, 37.88%, and 28.27%, respectively. Under combined stress, the HDDA treatment further enhanced the accumulation of osmotic regulating substances, increasing betaine, Pro, and SS levels by 223.30%, 87.91%, and 90.52%, respectively, compared with the HD-treated plants.

## 3. Discussion

### 3.1. Dopamine Alleviates Combined High Temperature and Drought Stress in Loquat Seedlings

Under the current background of global warming, high temperatures and drought have become the key factors constraining plant growth, development, yield, and quality. Root viability reflects the growth status and absorption function of plant roots [24]. Meanwhile, TBARS and EL are important indicators for assessing the extent of membrane lipid peroxidation and cell membrane integrity [25,26]. In this study, combined high temperature and drought stress impeded the physiological activities and growth of loquat seedlings, resulting in hampered root expansion, reduced root viability, declined cell membrane stability, leaf drooping, wilting, and decreased water content. These findings align with previous studies on tomatoes [27] and potatoes [28]. As a bioactive molecule with both signal transduction and antioxidant capabilities, dopamine can enhance a plant’s resistance to abiotic stress [19]. In this study, after dopamine treatment, the wilting severity of loquat seedlings was alleviated, and improvements were observed in the water content, root volume, surface area, viability and cell membrane stability of the seedlings. These findings are consistent with previous results in apples [22,29]. This demonstrates that dopamine can effectively enhance the tolerance of loquat seedlings to combined high temperature and drought stress and mitigate stress-induced damage. The mechanism might involve dopamine’s ability to enhance root oxidative capacity and increase the viscosity and elasticity of root tip cells, which stimulates root elongation [19,20], thereby improving root viability. Additionally, it may be related to dopamine-mediated photosynthesis, ROS homeostasis, and osmotic balance.

### 3.2. Physiological Mechanism of Dopamine’s Alleviation of Combined High Temperature and Drought Stress in Loquat Seedlings

Photosynthesis serves as the foundation of plant growth, providing the primary energy source and organic matter for plants. Its efficiency depends on the capture and conversion of light energy [30]. Chlorophyll and carotenoids are the key pigments responsible for this capture–conversion process [31]. In this study, combined high temperature and drought stress led to a significant decrease in photosynthetic pigment content, as well as reductions in *T*r and *G*s, ultimately causing a decline in *P*n. This phenomenon occurred because high temperature inhibits key enzyme activity in chlorophyll synthesis, thereby impeding chlorophyll production [32]. Additionally, in this study, combined stress reduced the root vitality in loquat seedlings. This reduction weakened mineral nutrient absorption (e.g., nitrogen) and diminished substrate supply for chlorophyll synthesis [33]. Consequently, these factors synergistically exacerbated chlorophyll loss. This photosynthetic impairment resulted in photosynthetic reaction center dysfunction, impaired electron transport, and energy deficits that compromised CO_2_ utilization [34,35], thereby reducing *P*n. Here, *P*n decreased while *C*i increased, indicating that a non-stomatal limitation was the cause of *P*n decline [36]. After dopamine treatment, the loquat seedlings showed significant increases in photosynthetic pigment content, *T*r, and *G*s, along with stabilized *C*i levels, thus resulting in a marked enhancement of *P*n. This improvement can be attributed to three synergistic actions of dopamine: (1) It boosts root viability, facilitating the uptake of mineral nutrients (e.g., nitrogen) to provide sufficient substrates for chlorophyll synthesis [37]. (2) It upregulates the activity of key chlorophyll synthesis enzymes and directly scavenges ROS, thereby reducing oxidative damage to chlorophyll porphyrin rings [38,39]. (3) It modulates abscisic acid signaling to moderately open stomata, which elevates the *G*s and CO_2_ supply, ultimately enhancing photosynthetic efficiency [40].

A brief and mild episode of stress triggers the production of ROS, which in turn induces the activities of antioxidant enzymes such as SOD, POD, CAT, and APX. In contrast, prolonged and severe stress causes a sharp and substantial accumulation of ROS that surpasses the capacity of these antioxidant enzymes to alleviate oxidative damage, resulting in a diminished ROS-scavenging ability and ultimately leading to membrane lipid peroxidation injury [11]. In this study, a significant accumulation of ROS, including superoxide anion and H_2_O_2_, was observed in loquat seedlings under combined high temperature and drought stress, consistent with prior findings in tomatoes [27] and potatoes [28]. These results indicate that the loquat seedlings suffered severe membrane lipid peroxidation damage under these stress conditions. As a stress response, ROS activated the activities of SOD, POD, CAT, and APX, yet the seedlings’ scavenging capacity was insufficient, leading to massive ROS accumulation [41]. Similar results have been confirmed in watermelons [42]. Under combined high temperature and drought stress, dopamine further enhanced the activities of antioxidant enzymes in the loquat seedlings while reducing ROS accumulation. These results align with previous studies on drought stress in apple trees [22]. This phenomenon may be attributed to dopamine’s dual mechanisms: It acts as a signaling molecule to directly trigger the expression of antioxidant enzyme genes [29] and to modulate stress-responsive transcription factors such as NAC, ERF, WRKY [43], and bHLH [44], thereby boosting antioxidant enzyme activity. Additionally, dopamine serves as a water-soluble antioxidant that directly scavenges ROS [22]. These combined effects effectively improve the stress resistance of loquat seedlings.

Plants accumulate osmoregulatory substances such as betaine, Pro, and SS to prevent excessive cellular water loss, maintain turgor pressure, reduce osmotic potential, and thereby ensure normal functioning of physiological metabolic processes under stress conditions [45,46]. In this study, combined high temperature and drought stress induced an increase in the contents of betaine, Pro, and SS in the loquat seedlings, which aligns with the reported results for winter wheat [47]. This represents another adaptive mechanism of loquat seedlings to combined high temperature and drought stress. However, osmoregulatory substances exhibit only marginal increases under stress conditions, resulting in severe cellular dehydration and consequential plant injury [48]. Following dopamine treatment, the loquat seedlings exhibited substantially elevated levels of betaine, Pro, and SS compared with those exposed to combined stress. This enhancement of osmoregulatory capacity aligns with previous findings of other hormones in grapes [49] and walnuts [50]. One possible explanation is that dopamine enhances osmotic stress-related gene expression and/or activates key biosynthetic enzymes, facilitating sucrose and malic acid accumulation via the upregulation of SPS1 (sucrose phosphate synthase) and MDH (malate dehydrogenase) [22,51]. These mechanisms collectively elevate the content of osmoregulatory substances, thereby maintaining cellular water potential and mitigating stress-induced damage, ultimately improving the thermotolerance and drought tolerance in loquat seedlings.

## 4. Materials and Methods

### 4.1. Materials

The experiment was conducted at Sichuan Agricultural University in Wenjiang (30°43′ N, 103°52′ E), China. The loquat plants used were one-year-old ‘Da Wu Xing’ seedlings cultivated in pots (25 cm in diameter and height; one plant per pot). The potting substrate was prepared by mixing field soil and universal nutrient soil at a 1:1 volumetric ratio, with a pH of 6.69, organic matter content of 35.37 g kg^−1^, alkali-hydrolyzable nitrogen of 143.23 mg kg^−1^, available phosphorus of 33.14 mg kg^−1^, and available potassium of 238.65 mg kg^−1^.

### 4.2. Experimental Design

First, loquat plants that were disease-free, pest-free, and exhibited uniform growth were selected. They were placed in a GZX-180F-type light incubator (Lichen Instrument Technology Co., Ltd., Shanghai, China) (day/night temperatures: 28 °C/18 °C; light/dark cycle: 12 h/12 h; light intensity: 120 μmol photons m^−2^ s^−1^; relative humidity (RH): 60%) for pre-cultivation. After one week, the plants were divided evenly into two groups for pretreatment: (1) foliar application of 100 μmol L^−1^ of exogenous dopamine (the optimal concentration determined through preliminary experiments, see Supplementary Materials); (2) control group: distilled water spray. The solutions were applied as a foliar spray, evenly coating the leaves until a thin water film (without droplet formation) covered the leaf surface. Applications were repeated every 2 days for a total of 3 applications. Following pretreatment, half of the plants from each group were randomly subjected to combined high temperature and drought stress in an RQH-350-type artificial climate chamber (Jinghong Laboratory Equipment Co., Ltd., Shanghai, China) (day/night temperatures: 38 °C/28 °C; light/dark cycle: 12 h/12 h; light intensity: 120 μmol photons m^−2^ s^−1^; RH: 60%). The remaining plants were kept under control conditions in the GZX-180F-type light incubator (day/night temperatures: 28 °C/18 °C; light/dark cycle: 12 h/12 h; light intensity: 120 μmol photons m^−2^ s^−1^; RH: 60%).

Four treatments were set up in the study. (1) Control (CK): foliar spray with distilled water under 28 °C/18 °C (day/night); (2) dopamine (DA): foliar spray with 100 μmol L^−1^ of dopamine solution under 28 °C/18 °C (day/night); (3) high temperature and drought stress (HD): foliar spray with distilled water under 38 °C/28 °C (day/night); (4) high temperature, drought and dopamine treatment (HDDA): foliar spray with 100 μmol L^−1^ of dopamine solution under 38 °C/28 °C (day/night). The experimental design consisted of three replicates per treatment, with fifteen pots per replicate.

Before pretreatment began, all pots were fully irrigated, and the soil relative water content (RWC) was measured using an SM150T portable moisture sensor (Delta-T Devices Ltd., London, UK). For the HD and HDDA treatments, soil moisture was regulated via the natural water depletion method [52]. When the soil RWC decreased to 40–45% of field capacity, this moisture level was maintained for 10 days using gravimetric measurements. The CK and DA treatments were kept at 70–80% field capacity. After 10 days, the 2nd–4th fully expanded leaves (from the apex) were harvested for physiological analysis.

### 4.3. Determination of Morphological Parameters

Representative seedlings were randomly selected from each treatment group for photographic documentation. Three randomly selected seedlings per treatment were used for root morphology analysis. The root systems were gently rinsed to remove adherent soil and scanned using a WinRHIZO LA-S plant image analysis system (WinRHIZO Pro LA-S; Wanshen Detection Technology Co., Ltd., Hangzhou, China). The scanned images were analyzed to determine key root morphological parameters, including root length, root surface area, root volume, average root diameter, and number of root tips.

### 4.4. Determination of Tissue Water Content and Relative Water Content

Root and leaf samples were collected, washed, and blotted dry before measuring fresh weight (FW). The samples were then immersed in water for several hours. After blotting dry, they were re-weighed. This immersion–blotting–weighing cycle was repeated at 1 h intervals until the saturated weight (Wt) stabilized (i.e., consecutive measurements showed negligible differences, indicating full saturation). Subsequently, the samples were placed in an oven at 105 °C for 30 min to deactivate enzymes, then dried at 60 °C until constant weight was achieved. Dry weight (DW) was recorded. The tissue water content (TWC) and relative water content (RWC) were calculated using the following formulas:

TWC (%) = [(FW − DW)/FW] × 100.

RWC (%) = [(FW − DW)/(Wt − DW)] × 100.

### 4.5. Determination of Photosynthetic Parameters, Photosynthetic Pigment Content, and Root Vitality

The net photosynthetic rate (*P*n, unit: μmol m^−2^ s^−1^), transpiration rate (*T*r, unit: mmol m^−2^ s^−1^), stomatal conductance (*G*s, unit: mol m^−2^ s^−1^), and intercellular CO_2_ concentration (*C*i, unit: μmol mol^−1^) were measured on the second fully expanded leaf below the apical meristem using an LI-6800 portable photosynthesis system (LI-COR, Lincoln, NE, USA). Measurements were conducted between 9:00 and 11:30 a.m. under controlled conditions: leaf chamber temperature (20–25 °C), CO_2_ concentration (380–420 μmol mol^−1^, regulated via an integrated CO_2_ cylinder), relative humidity (60%), and photosynthetically active radiation (800 μmol photons m^−2^ s^−1^).

Chlorophyll and total carotenoid contents were spectrophotometrically quantified in fresh leaf tissue according to Lichtenthaler [53] with modifications. Leaf samples were homogenized in 80% (*v*/*v*) aqueous acetone under subdued light conditions. The homogenate was centrifuged at 10,000× *g* for 10 min (4 °C), and the supernatant was collected for spectral analysis. Absorbance measurements were performed at 663 nm, 646 nm, and 470 nm using a double-beam spectrophotometer (Cary 3500-type, Agilent Technologies, Inc. Santa Clara, CA USA).

Pigment concentrations were calculated using the following equations:

Total Chlorophyll (mg L^−1^) = 7.15 × A_663_ + 18.71 × A_646_

Total carotenoids (mg L^−1^) = [1000 × A_470_ − 1.82 × (12.25 × A_663_ − 2.79 × A_646_) − 85.02 × (21.50 × A_646_ − 5.10 × A_663_)]/198

The final pigment content was expressed per unit fresh weight:

Pigment content (mg g^−1^ FW) = calculated concentration × extract volume/sample fresh weight

Root vitality was quantified via the triphenyl tetrazolium chloride (TTC) reduction method [54]. Fresh root apical segments (0.5 g, 5 mm length) were incubated in 6 mL of reaction solution containing 0.4% (*w*/*v*) TTC in a 0.1 M phosphate buffer (pH 7.4) at 37 °C in complete darkness for 2 h. The reaction was terminated with 2 mL of 1 M H_2_SO_4_. Formazan was extracted in ethyl acetate (final volume of 10 mL) and centrifuged (10,000× *g*, 4 °C, 10 min). The absorbance of the supernatant was measured at 485 nm using ethyl acetate as the blank.

A formazan standard curve was generated by dissolving 10 mg of TTF (1,3,5-triphenylformazan) in 100 mL of ethyl acetate (100 μg mL^−1^ stock solution). Serial dilutions (0–100 μg mL^−1^) were prepared, and A_485_ was measured for each standard.

The TTC reduction capacity was calculated as follows:

Root vitality (μg g^−1^ h^−1^ FW) = formazan concentration from standard curve ×  extraction volume/(fresh weight × incubation time)

### 4.6. Evaluation of Membrane Damage

Lipid peroxidation was assessed by quantifying the thiobarbituric acid-reactive substances (TBARS) content using the thiobarbituric acid (TBA) method [55]. Fresh leaf tissue (0.5 g) was homogenized in 5% (*w*/*v*) trichloroacetic acid (TCA) and centrifuged at 4000× *g* for 10 min. The supernatant was reacted with 0.67% (*w*/*v*) TBA in a boiling water bath for 30 min. After rapid cooling, absorbance was measured at 450 nm, 532 nm, and 600 nm. The TBARS content was calculated as follows:

TBARS (μmol g^−1^ FW) = [6.45 × (A_532_ − A_600_) − 0.56 × A_450_] × extract volume/fresh weight

Membrane permeability was assessed via electrolyte leakage (EL) using the method of Ifeduba [56]. Uniform leaf discs (5 mm diameter) were excised with a cork borer (major veins excluded), rinsed in distilled water to remove surface electrolytes, and immersed in 10 mL of distilled water. Samples were incubated at 25 °C for 1 h with gentle shaking. Initial conductivity (C_1_) was measured using a conductivity meter. Discs were then boiled in the same solution for 10 min and cooled to 25 °C, and then final conductivity (C_2_) was recorded. EL was calculated as follows:

EL (%) = C_1_/C_2_ ×100.

### 4.7. Determination of ROS

The superoxide anion production rate was quantified using the hydroxylamine oxidation method [57]. Fresh leaf tissue (0.5 g) was homogenized in ice-cold phosphate-buffered saline (PBS) and centrifuged at 12,000× *g* for 20 min (4 °C). The supernatant (1 mL) was reacted with 1 mL of a hydroxylamine hydrochloride solution for 1 h at 25 °C. Subsequently, 1 mL of sulfanilamide and 1 mL of α-naphthylamine were added, followed by incubation at 25 °C for 30 min. Absorbance was measured at 530 nm. A standard curve was generated by preparing NaNO_2_ solutions (0–50 μM in PBS) and processing them identically to the samples. The superoxide anion production rate was calculated as follows:

Superoxide anion production rate (μmol g^−1^ min^−1^ FW) = (nitrite concentration from standard curve −  endogenous nitrite control) × extraction volume/(fresh weight × hydroxylamine incubation time)

The hydrogen peroxide (H_2_O_2_) content was measured using a commercial assay kit (Jiancheng Biotech Inc., Nanjing, China). Fresh leaf tissue (0.1 g) was homogenized in 1 mL of an ice-cold extraction buffer (Reagent 1) and centrifuged at 8000× *g* for 10 min (4 °C). The supernatant was collected. To 0.5 mL of the supernatant, 100 μL of a reaction initiator (Reagent 2) and 200 μL of a chromogenic agent (Reagent 3) were added. The mixture was centrifuged at 4000× *g* for 10 min at 25 °C. After carefully discarding the supernatant, the pellet was resuspended in 1 mL of a developer solution (Reagent 4) and incubated at 25 °C for 5 min. Absorbance was measured at 415 nm against a reagent blank. The H_2_O_2_ standard was used instead of the sample in the standard preparation group.

Calculate net absorbance: ΔA_Test_ = A_Test_ − A_Blank_ ΔA_Standard_ = A_Standard_ − A_Blank_

H_2_O_2_ (μmol g^−1^ FW) = (ΔA_Test_/ΔA_Standard_)/fresh weight

### 4.8. Determination of Antioxidant Enzyme Activities

Superoxide dismutase (SOD) activity was determined with the nitroblue tetrazolium (NBT) photochemical reduction method [58]. Fresh leaf tissue (0.5 g) was homogenized in 5 mL of an ice-cold phosphate buffer (50 mM, pH 7.8) and centrifuged at 12,000× *g* for 15 min (4 °C). The supernatant (crude enzyme extract) was kept on ice. The reaction mixtures contained 1.5 mL of a phosphate buffer (50 mM, pH 7.8), 0.3 mL of L-methionine (13 mM), 0.3 mL of NBT (75 μM), 0.3 mL of EDTA-Na_2_ (0.1 mM), 0.3 mL of riboflavin (2 μM), 50 μL of an enzyme extract, and 0.25 mL of distilled water. The controls replaced the enzyme extract with a phosphate buffer. Tubes were illuminated (75 μmol photons m^−2^ s^−1^, 30 min), with one dark control wrapped in foil. Absorbance at 560 nm was measured. One unit of SOD activity (U) was defined as the amount of enzyme required to cause a 50% inhibition of NBT photochemical reduction relative to the light-exposed control. SOD activity was calculated using the following equation:

SOD activity (U g^−1^ FW) = [(absorbance of light-exposed control − absorbance of sample) × reaction  volume]/[0.5 × absorbance of light-exposed control × fresh weight × volume of enzyme extract in reaction]

Peroxidase (POD) activity was determined by guaiacol oxidation [59]. Fresh leaf tissue (0.5 g) was homogenized in an ice-cold phosphate buffer (50 mM, pH 7.0) and centrifuged at 10,000 × g for 15 min (4 °C). The reaction mixtures contained 700 μL of a phosphate buffer (0.1 M, pH 7.0), 100 μL of guaiacol (20 mM), and 100 μL of an enzyme extract. The reactions were initiated with 100 μL of H_2_O_2_ (10 mM), and absorbance at 470 nm was recorded every 10 s for 1 min using a spectrophotometer. POD activity was calculated from the initial linear rate of change (ΔA_470_). One unit of POD activity (U) was defined as a change of 0.01 per minute in A_470_.

POD activity (U g^−1^ FW) = ΔA_470_ × reaction volume/(0.01 × fresh weight × reaction time × volume of  enzyme extract in reaction)

Catalase (CAT) activity was measured by H_2_O_2_ decomposition at 240 nm [58]. Fresh leaf tissue (0.5 g) was homogenized in an ice-cold phosphate buffer (50 mM, pH 7.0) containing 1 mM of EDTA and 1% (*w*/*v*) polyvinylpyrrolidone (PVP), followed by centrifugation at 12,000 × g for 15 min (4 °C). A supernatant (50 μL) was added to 1.95 mL of a phosphate buffer (50 mM, pH 7.0) in a quartz cuvette. The reactions were initiated with 1 mL of H_2_O_2_ (15 mM), and absorbance decrease at 240 nm (ΔA_240_) was recorded for 1 min. CAT activity was derived from the initial reaction rate (ΔA_240_). One unit of CAT activity (U) was defined as a change of 0.1 per minute in A_240_.

CAT activity (U g^−1^ FW) = ΔA_240_ × reaction volume/(0.1 × fresh weight ×  reaction time × volume of enzyme extract in reaction)

Ascorbate peroxidase (APX) activity was quantified via ascorbate oxidation at 290 nm [58]. Fresh leaf tissue (0.5 g) was homogenized in an ice-cold potassium phosphate buffer (50 mM, pH 7.0) containing 1 mM of ascorbate, 1 mM of EDTA, and 1% PVP, then centrifuged at 12,000× *g* for 15 min (4 °C). The reaction mixtures (1 mL final volume) contained 50 mM of a phosphate buffer (pH 7.0), 0.5 mM of ascorbate, and 0.1 mM of EDTA. After adding 50 μL of a supernatant, the reactions were initiated with 0.1 mL of H_2_O_2_ (1 mM), and absorbance decrease at 290 nm (ΔA_290_) was recorded for 1 min. APX activity was calculated from the initial reaction rate (ΔA_290_).

APX activity (μmol g^−1^ min^−1^ FW) = ΔA_290_ × reaction volume/(2.8 × 10^3^ × fresh weight ×  cuvette path length × reaction time × volume of enzyme extract in reaction)

### 4.9. Analysis of Osmotic Regulating Substances

The proline (Pro) content was determined using the acidic ninhydrin method [60]. Fresh leaf tissue (0.5 g) was homogenized in 10 mL of 3% (*w*/*v*) sulfosalicylic acid and centrifuged at 10,000× *g* for 10 min. A 2 mL aliquot of the supernatant was reacted with 2 mL of an acid–ninhydrin reagent (1.25 g of ninhydrin dissolved in 30 mL of glacial acetic acid and 20 mL of 6 M H_3_PO_4_) and 2 mL of glacial acetic acid. The mixture was incubated at 100 °C for 1 h, cooled on ice, and extracted with 4 mL of toluene. After phase separation (10 min), the absorbance of the toluene layer was measured at 520 nm. A standard curve was generated concurrently using L-proline solutions (0–20 μg mL^−1^) processed identically to the samples. The absorbance values were plotted against proline concentration to derive a linear regression equation (y = ax + b). The proline content was calculated as follows:

Proline (μg g^−1^ FW) = (proline concentration determined from the standard curve ×  volume of toluene used for extraction)/fresh weight

The betaine content was analyzed using a commercial kit (Shanghai Yaji Biotechnology Co., Ltd., Shanghai, China). Dried leaf tissue (40 mg) was extracted in 1.6 mL of distilled water (60 °C, 30 min, shaking), followed by centrifugation at 10,000× *g* (25 °C, 10 min). A 300 μL aliquot of a supernatant (Test) and 300 μL of distilled water (Blank) were transferred to separate reaction tubes. To each tube, 500 μL of a precipitation reagent (Reagent I) was added. After vortexing, tubes were incubated at 4 °C for 2 h. Following centrifugation (12,000× *g*, 10 min, 25 °C), the supernatants were discarded. The pellets were washed with 500 μL of washing solution (Reagent II; 80% ethanol/ether mixture) and re-centrifuged under identical conditions, and then the supernatants were completely removed. The residual solvent was evaporated for 15 min in a fume hood. The pellets were dissolved in 300 μL color developer (Reagent III) by vigorous vortexing. Aliquots (200 μL) were transferred to a 96-well plate, and absorbance was measured at 525 nm using a microplate reader pre-equilibrated to 25 °C. A standard curve (0–200 μg mL^−1^ betaine) was processed identically to samples. The betaine content was calculated as follows:

Betaine (mg g^−1^ DW) = [betaine concentration from standard curve × extraction volume]/dry weight

Soluble sugars (SS) were assessed with the anthrone colorimetric method [61]. Fresh tissue (0.5 g) was extracted by boiling in 10 mL of distilled water for 30 min. After centrifugation (3000× *g*, 10 min), the pellet was re-extracted with 10 mL boiling water. Combined supernatants were brought to a final volume of 50 mL with distilled water. For the colorimetric reaction, 0.5 mL of extract was mixed with 1.5 mL of distilled water, 0.5 mL of ice-cold anthrone reagent (0.2% *w*/*v* in concentrated H_2_SO_4_), and 5 mL of concentrated H_2_SO_4_. The mixture was vortexed immediately and heated at 100 °C for exactly 1 min. After rapid cooling to room temperature, the absorbance measured at 630 nm. A standard curve was generated by preparing glucose solutions (0–200 µg mL^−1^) and processing them identically to the samples. Soluble sugar concentration was determined from the standard curve using the formula:

SS (mg g^−1^ FW) = (sugar concentration from standard curve × extract volume)/ (volume of extract assayed × fresh weight)

### 4.10. Statistical Analysis

All data are presented as the means ± SE of three independent replicates. The data were analyzed with a one-way ANOVA using SPSS 25.0 (IBM, Inc., Armonk, NY, USA). Tukey’s multiple comparison test was used to detect differences between treatments at *p* < 0.05. All figures were plotted using Origin 9.0 (OriginLab Corporation, Northampton, MA, USA).

## 5. Conclusions

Dopamine can enhance the tolerance of loquat seedlings to combined high temperature and drought stress. The primary mechanisms of action are as follows: (1) Dopamine improves the photosynthetic efficiency of loquat seedlings by maintaining photosynthetic pigment levels and increasing stomatal conductance. (2) It mitigates membrane lipid peroxidation damage by boosting the activity of antioxidant enzymes in the seedlings, thus effectively scavenging the ROS generated within the plants. (3) Dopamine promotes the accumulation of osmotic substances to regulate osmotic potential, thereby enhancing the seedlings’ high temperature and drought tolerance. In addition, dopamine may regulate this tolerance in loquat seedlings either through its own metabolites or by regulating other plant hormone levels, the specific mechanisms for these pathways requiring further investigation.

## Figures and Tables

**Table 1:** Root morphological parameters of loquat [*Eriobotrya japonica* (Thunb.) Lindl.] seedlings.

Treatment	Length (cm)	Surface Area (cm^2^)	Volume (cm^3^)	Mean Diameter (mm)	Number of Tips
CK	1257.432 ± 84.211 ^ab^	712.033 ± 24.115 ^a^	70.036 ± 5.004 ^a^	1.925 ± 0.157 ^a^	214.775 ± 14.467 ^a^
DA	1311.926 ± 55.772 ^a^	728.285 ± 23.368 ^a^	72.069 ± 4.806 ^a^	2.084 ± 0.104 ^a^	225.817 ± 12.445 ^a^
HD	801.002 ± 67.605 ^c^	500.624 ± 26.007 ^b^	41.992 ± 8.427 ^c^	1.346 ± 0.112 ^c^	140.582 ± 6.404 ^c^
HDDA	1164.553 ± 57.623 ^b^	544.464 ± 45.018 ^b^	54.955 ± 4.720 ^b^	1.647 ± 0.153 ^b^	163.231 ± 4.940 ^b^

Note: Data are presented as the means ± SE (n = 3). Different letters following the values in the same column indicate significant differences (*p* < 0.05) among different treatments based on Tukey’s HSD test. CK: control, foliar spray with distilled water; DA: foliar spray with dopamine solution; HD: combined high temperature and drought stress, foliar spray with distilled water; HDDA: combined high temperature and drought stress, foliar spray with dopamine solution.

**Table 2:** The tissue water content and relative water content in roots and leaves of loquat [*Eriobotrya japonica* (Thunb.) Lindl.] seedlings.

Treatment	TWC (%)		RWC (%)	
	Leaf	Root	Leaf	Root
CK	75.152 ± 1.193 ^a^	70.963 ± 3.795 ^a^	82.253 ± 3.613 ^a^	88.078 ± 2.668 ^a^
DA	79.910 ± 5.591 ^a^	73.195 ± 5.264 ^a^	84.482 ± 2.958 ^a^	86.825 ± 4.764 ^a^
HD	46.584 ± 4.701 ^c^	57.375 ± 3.073 ^c^	56.921 ± 6.430 ^c^	65.515 ± 2.173 ^c^
HDDA	61.558 ± 5.589 ^b^	65.320 ± 2.882 ^b^	67.440 ± 2.351 ^b^	77.424 ± 4.753 ^b^

Note: Data are presented as the means ± SE (n = 3). Different letters following the values in the same column indicate significant differences (*p* < 0.05) among different treatments based on Tukey’s HSD test. CK: control, foliar spray with distilled water; DA: foliar spray with dopamine solution; HD: combined high temperature and drought stress, foliar spray with distilled water; HDDA: combined high temperature and drought stress, foliar spray with dopamine solution.

**Table 3:** Photosynthetic pigments and photosynthetic parameters of loquat [*Eriobotrya japonica* (Thunb.) Lindl.] seedlings.

Treatment	Carotenoid Content (mg g^−1^ FW)	Chlorophyll Content (mg g^−1^ FW)	*P*n (μmol m^−2^ s^−1^)	*T*r (mmol m^−2^ s^−1^)	*G*s (mol m^−2^ s^−1^)	*C*i (μmol mol^−1^)
CK	0.083 ± 0.009 ^b^	0.662 ± 0.014 ^b^	14.681 ± 0.847 ^a^	3.900 ± 0.390 ^a^	0.134 ± 0.013 ^a^	204.858 ± 8.882 ^c^
DA	0.081 ± 0.003 ^b^	0.704 ± 0.005 ^a^	15.766 ± 0.725 ^a^	4.201 ± 0.333 ^a^	0.159 ± 0.009 ^a^	213.073 ± 1.123 ^c^
HD	0.067 ± 0.004 ^c^	0.499 ± 0.021 ^d^	3.770 ± 0.234 ^c^	1.343 ± 0.110 ^c^	0.030 ± 0.011 ^c^	251.606 ± 8.018 ^a^
HDDA	0.099 ± 0.007 ^a^	0.612 ± 0.024 ^c^	8.353 ± 0.240 ^b^	3.147 ± 0.376 ^b^	0.081 ± 0.019 ^b^	232.442 ± 1.375 ^b^

Note: Data are presented as the means ± SE (n = 3). Different letters following the values in the same column indicate significant differences (*p* < 0.05) among different treatments based on Tukey’s HSD test. CK: control, foliar spray with distilled water; DA: foliar spray with dopamine solution; HD: combined high temperature and drought stress, foliar spray with distilled water; HDDA: combined high temperature and drought stress, foliar spray with dopamine solution.

## Data Availability

The original contributions presented in this study are included in the article/Supplementary Materials. Further inquiries can be directed to the corresponding author.

## Supplementary Materials

The following supporting information can be downloaded at: https://www.mdpi.com/article/10.3390/plants14172650/s1, Figure S1: Root viability and SPAD in loquat seedlings; Figure S2: TBARS content and EL in loquat seedlings; Figure S3: Photosynthetic parameters in loquat seedlings.

## Abbreviations

The following abbreviations are used in this manuscript:

DA	Dopamine
ROS	Reactive oxygen species
TBARS	Thiobarbituric acid-reactive substances
EL	Electrolyte leakage
RH	Relative humidity
SOD	Superoxide dismutase
CAT	Catalase
POD	Peroxidase
APX	Ascorbate peroxidase
HD	High temperature and drought
HDDA	Dopamine was applied under high temperature and drought stress
Pn	Net photosynthetic rate
Tr	Transpiration rate
Gs	Stomatal conductance
Ci	Intercellular CO_2_ concentration
Pro	Proline
SS	Soluble sugar
SPS	Sucrose phosphate synthase
MDH	Malate dehydrogenase
RWC	Relative water content
PAR	Photosynthetically active radiation
