# Supplementary material for: Exogenous Dopamine Alleviates Combined High Temperature and Drought Stress in Loquat [Eriobotrya japonica (Thunb.) Lindl.] Seedlings: Improvements in Photosynthetic Efficiency, Oxidative Damage and Osmotic Regulation
Source: Plants (Basel). 2025 Aug 26;14(17):2650. doi: 10.3390/plants14172650 (PMC12429963; doi:10.3390/plants14172650)
Supplement: Supplementary file 1 [file plants-14-02650-s001.zip › plants-3800791-supplementary.pdf]

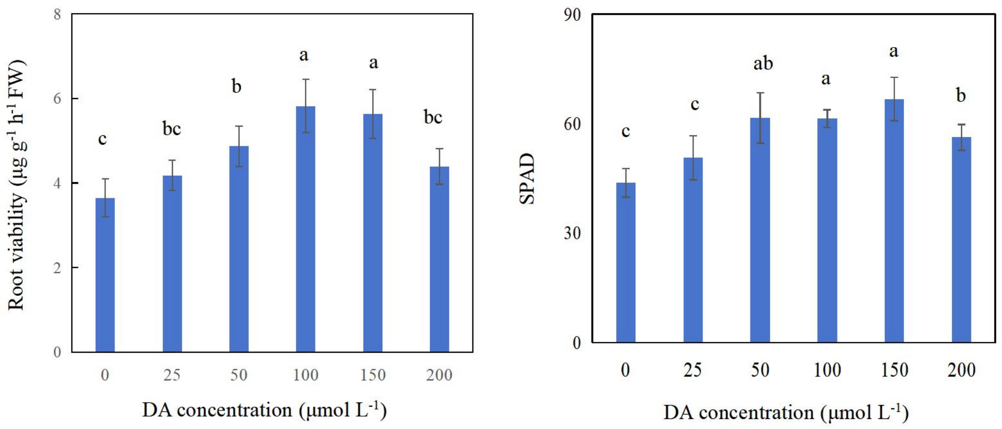

Figure S1. Root viability and SPAD in loquat [*Eriobotrya japonica* (Thunb.) Lindl.] seedlings

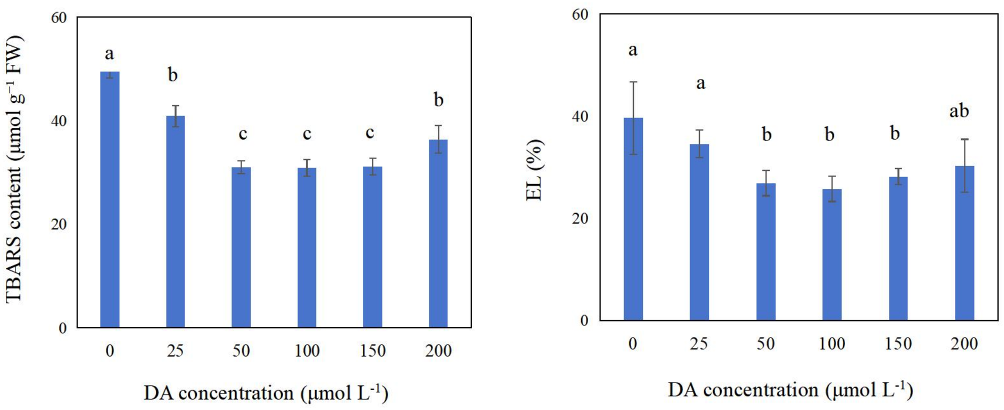

Figure S2. TBARS content and EL in loquat [*Eriobotrya japonica* (Thunb.) Lindl.] seedlings

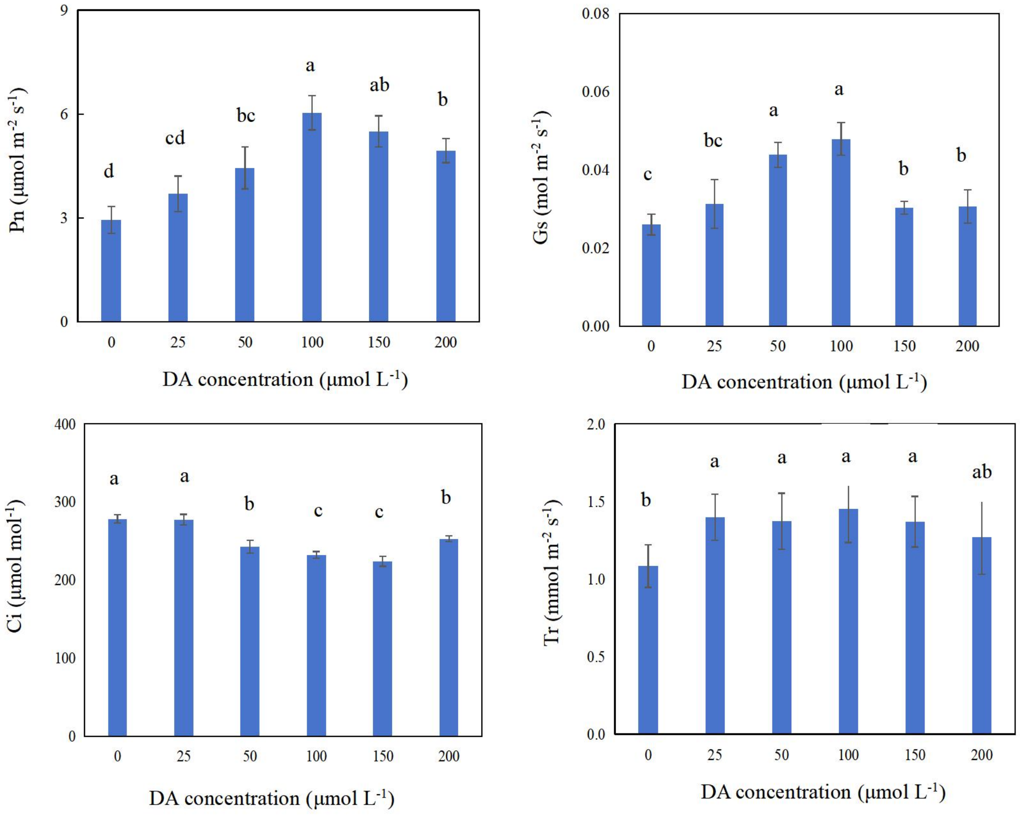

Figure S3. Photosynthetic parameters in loquat [*Eriobotrya japonica* (Thunb.) Lindl.] seedlings
